# Volatile anaesthetics enhance the metastasis related cellular signalling including CXCR2 of ovarian cancer cells

**DOI:** 10.18632/oncotarget.8304

**Published:** 2016-03-23

**Authors:** Masae Iwasaki, Hailin Zhao, Tanweer Jaffer, Sandeep Unwith, Laura Benzonana, Qingquan Lian, Atsuhiro Sakamoto, Daqing Ma

**Affiliations:** ^1^ Anaesthetics, Pain Medicine and Intensive Care, Department of Surgery and Cancer, Faculty of Medicine, Imperial College London, Chelsea & Westminster Hospital, London, UK; ^2^ Department of Anaesthesiology, Nippon Medical School, Tokyo, Japan; ^3^ Department of Anesthesiology, Second Affiliated Hospital, Wenzhou Medical University, Wenzhou, China

**Keywords:** isoflurane, ovarian cancer, tumour metastasis, CXCR2

## Abstract

The majority of ovarian cancer patients relapse after surgical resection. Evidence is accumulating regarding the role of surgery in disseminating cancer cells; in particular anaesthesia may have an impact on cancer re-occurrence. Here, we have investigated the metastatic potential of volatile anaesthetics isoflurane, sevoflurane and desflurane on ovarian cancer cells.

Human ovarian carcinoma cells (SKOV3) were exposed to isoflurane (2%), sevoflurane (3.6%) or desflurane (10.3%) for 2 hours. Metastatic related gene expression profiles were measured using the Tumour Metastasis PCR Array and qRT-PCR. Subsequently vascular endothelial growth factor A (VEGF-A), matrix metalloproteinase 11 (MMP11), transforming growth factor beta-1 (TGF-β1) and chemokine (C-X-C motif) receptor 2 (CXCR2) proteins expression were determined using immunofluorescent staining. The migratory capacities of SK-OV3 cells were assessed with a scratch assay and the potential role of CXCR2 in mediating the effects of volatile anaesthetics on cancer cell biology were further investigated with CXCR2 knockdown by siRNA.

All three volatile anaesthetics altered expression of 70 out of 81 metastasic related genes with significant increases in VEGF-A, MMP-11, CXCR2 and TGF-β genes and protein expression with a magnitude order of desflurane (greatest), sevoflurane and isoflurane. Scratch analysis revealed that exposure to these anesthetics increased migration, which was abolished by CXCR2 knockdown.

Volatile anaesthetics at clinically relevant concentrations have strong effects on cancer cell biology which in turn could enhance ovarian cancer metastatic potential. This work raises the urgency for further *in vivo* studies and clinical trials before any conclusions can be made in term of the alteration of clinical practice.

## INTRODUCTION

The death rate from ovarian cancer in the United States is more than double that of any other gynaecological malignancy [1, 2]. The poor prognosis is not only due to the aggressive nature of this disease but also because metastases are often present at the time of diagnosis or surgery [3]. It is thought that perioperative factors may contribute to cancer recurrence [4]. Surgical procedures such as biopsy and resection have been reported to disseminate cancer cells into the circulation and surrounding tissues [5] and many studies have reported that general anaesthesia dampens immune function, which is required to eliminate cancer cells [3, 6, 7].

The effect of general anaesthetics on healthy cells or tissues in the micro-environment have been investigated for many years and both volatile and intravenous agents have been shown to alter miRNA, mRNA and protein expressions [7, 8]. A variety of anaesthetics are used for cancer resection without their direct cellular effects on cancer cells being known. Recent clinical evidence has indicated that the choice of anaesthesia and application technique could potentially change the long-term prognosis of cancer patients. It has been shown that, compared to general anaesthesia, epidural anaesthesia for surgery to resect colonic cancer is associated with improved survival [9]. The latest study indicated an association between certain inhalational anesthetics and ovarian cancer outcomes [10] It was also reported that mortality was increased for patients with melanoma when receiving general, rather than local, anesthesia for the surgical removal of the tumour [11]. Despite the routine use of a variety of anaesthetics in cancer surgery, little research has been done to date on cancer cells and the molecular mechanism of how cancer cells interact with inhalational anaesthetics gas remains largely unknown. It has been shown that sevoflurane increases breast cancer cell proliferation in vitro[12]. It is therefore crucial to investigate the possible effect of anaesthetics on cancer cells.

In this study, our aim is to explore whether commonly used volatile anaesthetic agents isoflurane, sevoflurane and desflurane affect tumour metastasis related genes and hence proteins in ovarian cancer cells and to further investigate whether the effects (if any) could enhance cancer cell migration potential.

## RESULTS

### Effects of volatile anaesthetics on metastatic gene expressions in SK-OV-3 cells

**Figure 1:**
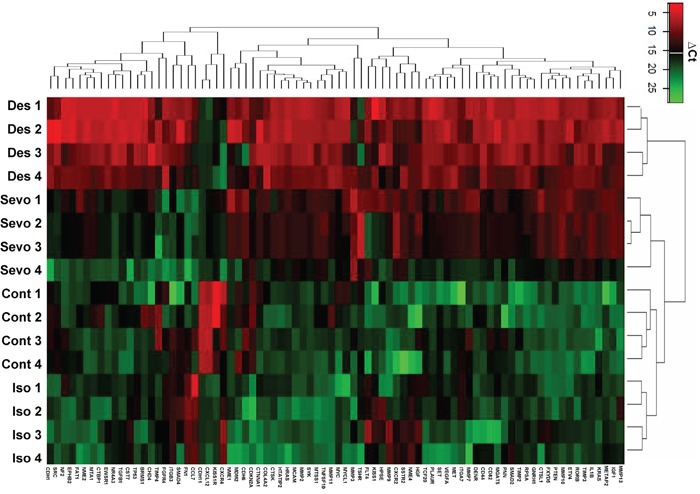
Isoflurane, Sevoflurane and Desflurane alter mRNA expression levels of tumour metastasis genes shown by array analysis SK-OV3 cells were treated with air (N_2_) or 2% Isoflurane (Iso) or 3.6% sevoflurane or 10.3% Desflurane for 2 hours, and then recovered in the normal cell incubator for up to 24 hours. Six hours after exposure analysis of the tumour metastasis PCR array was carried out. Unsupervised hierarchical cluster analysis using Euclidean distance from TaqMan low-density arrays. Gas treatment induced changes in the expression of 70 out of 81 mRNAs relative to the controls and, in comparison to sevoflurane and isoflurane, desflurane led to greater increases in mRNA (N=4). All data is relative to endogenous control, β-Actin. Red and green colours indicate relatively high and low expression, respectively.

**Figure 2:**
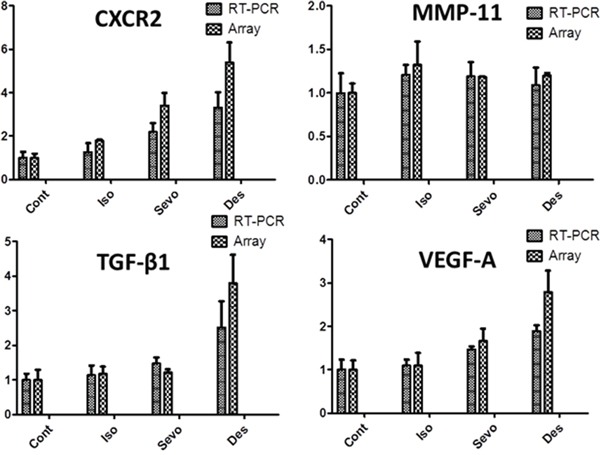
Isoflurane, Sevoflurane and Desflurane alter mRNA expression levels of tumour metastasis genes shown by RT-PCR SK-OV3 cells were treated with air (N_2_) or 2% isoflurane (Iso) or 3.6% sevoflurane (Sevo) or 10.3% desflurane (Des) for 2 hours, and then recovered in the normal cell incubator for up to 24 hours. Six hours after exposure analysis of the tumour metastasis PCR array was carried out. Results obtained from the tumour metastasis PCR array and RT-PCR analysis are well correlated. All data is displayed as relative to the endogenous control, β-Actin (n = 4). Data are expressed as mean ± SD. NC: naïve control. Iso: isoflurane, Sevo: sevoflurane, Des: desflurane.

The tumour metastasis PCR array enabled the analysis of 81 mRNAs including 5 endogenous control candidates after 6 hours of gas exposure (Figure 1). All three gases induced changes in the mRNA expression level of 70 out of 81 mRNAs (Table 1), but different inhalational anaesthetics had distinct effects on changes in the gene expression profile (Table 2). In particular, changes induced by desflurane were different to those seen with exposure to isoflurane and sevoflurane (Figure 1), with desflurane leading to greater increases in mRNA. In order to validate these array results, qRT-PCR was performed on a proportion of the 81 mRNAs in the array analysis (Figure 2). The array analysis and the qRT-PCR results are well correlated. For example; VEGF-A mRNA expressions seen with qRT-PCR were 1.00 ± 0.23 (control), 1.10 ± 0.14 (isoflurane), 1.47 ± 0.07 (sevoflurane) and 1.89 ± 0.13 (desflurane), and those from array analysis were 1.00 ± 0.21 (control), 1.09 ± 0.29 (isoflurane), 1.66 ± 0.28 (sevoflurane) and 2.78 ± 0.49 (desflurane) relative to the control. In addition, this analysis revealed that four of the mRNA (CXCR2, TGF-β, VEGF-A and MMP11) were significantly up-regulated and thus they were chosen for further investigation of protein expression.

**Table 1:** Change of the expression of the metastatic genes in all the volatile anesthetics treated group (Iso = Isoflurane, Sevo = Sevoflurane, Des = Desflurane) compared with the control group

Table 1A: Decrease in all anaesthesia group							
Assay	Relative change				P-value (Q-value)		
	Cont	Iso	Sevo	Des	Cont-Iso	Cont-Sevo	Cont-Des
CDH6	1.000±0.160	0.001±0.000	0.001±0.000	0.001±0.000	<0.001(<0.001)	<0.001(<0.001)	<0.001(<0.001)
CXCR4	1.000±0.200	0.057±0.016	0.106±0.026	0.068±0.014	<0.001(<0.001)	<0.001(<0.001)	<0.001(<0.001)

Table 1B: Increased in all anaesthesia group							
Assay	Relative change				P-value (Q-value)		
	Cont	Iso	Sevo	Des	Cont-Iso	Cont-Sevo	Cont-Des
NME2	1.000±0.278	2.081±0.265	1.951±0.494	2.624±0.385	0.002(0.005)	0.005(0.012)	<0.001(0.001)
SSTR2	1.000±0.434	2.072±0.506	2.770±0.259	3.619±0.922	0.02(0.038)	0.002(0.005)	<0.001(0.001)
HPSE	1.000±0.650	1.981±0.510	2.301±0.563	2.567±0.325	0.029(0.051)	0.010(0.021)	0.004(0.010)
TIMP3	1.000±0.191	1.580±0.151	3.004±0.358	5.103±0.589	0.002(0.005)	<0.001(<0.001)	<0.001(<0.001)

**Table 2:** Change of the expression of the metastatic genes in the single volatile anesthetics treated group (Iso = Isoflurane, Sevo = Sevoflurane, Des = Desflurane) compared with the control group

Table 2A: Increased in Iso group only							
Assay	Relative change				P-value (Q-value)		
	Cont	Iso	Sevo	Des	Cont-Iso	Cont-Sevo	Cont-Des
CCL7	1.000±0.255	1.606±0.347	0.835±0.105	1.256±0.197	0.016(0.032)	0.625	0.314

Table 2B: Decreased in Iso group only							
Assay	Relative change				P-value (Q-value)		
	Cont	Iso	Sevo	Des	Cont-Iso	Cont-Sevo	Cont-Des
CHD4	1.000±0.133	0.331±0.060	0.741±0.105	1.124±0.166	<0.001(<0.001)	0.067	0.712

Table 2C: Increased in Des group only							
Assay	Relative change				P-value (Q-value)		
	Cont	Iso	Sevo	Des	Cont-Iso	Cont-Sevo	Cont-Des
CDH1	1.000±0.504	0.746±0.346	1.443±0.235	4.274±1.809	0.785	0.511	0.003(0.008)
TGFB1	1.000±0.292	1.187±0.292	1.217±0.091	2.788±0.827	0.618	0.451	<0.001(<0.001)
EWSR1	1.000±0.124	0.869±0.215	1.217±0.208	3.664±1.386	0.820	0.731	<0.001(0.001)
NME1	1.000±0.186	0.996±0.228	1.499±0.304	3.155±0.815	1.000	0.116	<0.001(<0.001)
TP53	1.000±0.233	1.063±0.325	1.055±0.158	3.347±0.861	0.994	0.981	<0.001(0.001)
TCF20	1.000±0.255	0.925±0.384	1.276±0.118	3.054±0.280	0.970	0.440	<0.001(0.001)
SMAD2	1.000±0.207	1.315±0.158	1.357±0.213	2.931±0.404	0.146	0.103	<0.001(<0.001)
NME4	1.000±0.105	1.015±0.025	0.868±0.055	2.208±0.436	0.996	0.425	<0.001(<0.001)
PLAUR	1.000±0.363	1.008±0.215	1.390±0.128	2.206±0.358	0.991	0.202	0.003(0.008)
SRC	1.000±0.098	0.678±0.065	0.783±0.073	2.114±0.789	0.059	0.318	0.001(0.003)
CD82	1.000±0.287	1.748±0.304	1.574±0.564	2.967±1.669	0.139	0.314	0.007(0.015)
HTATIP2	1.000±0.094	0.827±0.236	1.530±0.271	2.731±0.661	0.529	0.090	<0.001(0.001)
FGFR4	1.000±0.225	1.079±0.249	1.169±0.181	2.765±0.524	0.963	0.688	<0.001(<0.001)
CTBP1	1.000±0.210	1.380±0.138	1.128±0.325	2.427±0.585	0.256	0.940	0.001(0.004)
CTNNA1	1.000±0.292	0.685±0.184	0.832±0.127	2.413±0.560	0.174	0.770	0.001(0.003)
CD44	1.000±0.136	0.886±0.234	1.293±0.146	2.395±0.144	0.634	0.183	<0.001(<0.001)
ITGA7	1.000±0.463	1.237±0.386	1.581±0.372	2.237±0.289	0.695	0.209	0.020(0.040)
METAP2	1.000±0.128	0.853±0.256	1.230±0.254	2.153±0.483	0.675	0.652	0.003(0.004)
FN1	1.000±0.271	1.099±0.077	0.681±0.159	2.042±0.353	0.837	0.093	0.001(0.004)
CST7	1.000±0.128	0.935±0.102	1.238±0.199	2.000±0.209	0.884	0.151	<0.001(<0.001)
SET	1.000±0.214	0.953±0.216	1.112±0.162	1.905±0.261	0.985	0.835	0.003(0.007)
MYCL1	1.000±0.173	0.944±0.217	1.064±0.165	1.825±0.417	0.966	0.969	0.006(0.014)
MMP9	1.000±0.174	0.816±0.111	1.133±0.191	1.814±0.313	0.390	0.735	0.002(0.005)
FLT4	1.000±0.098	0.850±0.108	1.179±0.299	1.790±0.232	0.503	0.607	0.001(0.004)
MTSS1	1.000±0.246	0.903±0.157	1.133±0.020	1.786±0.099	0.847	0.576	0.001(0.003)
MMP7	1.000±0.133	1.354±0.417	1.352±0.199	1.749±0.196	0.054	0.095	0.002(0.005)
CTSK	1.000±0.160	0.826±0.154	1.199±0.151	1.621±0.402	0.476	0.521	0.017(0.035)
CDH11	1.000±0.058	0.761±0.109	0.800±0.177	1.589±0.379	0.196	0.293	0.023(0.044)
HRAS	1.000±0.107	1.146±0.268	1.162±0.262	1.588±0.315	0.830	0.765	0.033(0.058)
MYC	1.000±0.127	0.727±0.121	1.164±0.200	1.563±0.283	0.066	0.599	0.012(0.025)
ETV4	1.000±0.090	0.935±0.151	1.313±0.223	1.542±0.218	0.894	0.110	0.007(0.017)
SMAD4	1.000±0.220	1.056±0.206	0.941±0.147	1.519±0.258	0.973	0.986	0.045(0.075)
COL4A2	1.000±0.250	0.994±0.081	1.041±0.171	1.486±0.213	0.999	0.971	0.027(0.050)
NF2	1.000±0.045	0.884±0.125	1.340±0.298	1.478±0.315	0.712	0.162	0.040(0.069)

Table 2D: Decreased in Des group only							
Assay	Relative change				P-value (Q-value)		
	Cont	Iso	Sevo	Des	Cont-Iso	Cont-Sevo	Cont-Des
EPHB2	1.000±0.224	0.942±0.182	0.656±0.130	0.586±0.122	0.980	0.061	0.016(0.032)

Table 2E: Decreased in Iso group and increased in Sevo and Des group							
Assay	Relative change				P-value (Q-value)		
	Cont	Iso	Sevo	Des	Cont-Iso	Cont-Sevo	Cont-Des
MTA1	1.000±0.240	0.616±0.092	1.775±0.259	2.823±0.623	0.030(0.054)	0.007(0.016)	<0.001(<0.001)

Table 2F: Increased in Sevo and Des group							
Assay	Relative change				P-value (Q-value)		
	Cont	Iso	Sevo	Des	Cont-Iso	Cont-Sevo	Cont-Des
CXCR2	1.000±0.185	1.792±0.059	2.918±0.235	4.902±1.073	0.139	<0.001(<0.001)	<0.001(<0.001)
PTEN	1.000±0.198	1.118±0.309	3.357±0.615	5.566±1.147	0.922	<0.001(<0.001)	<0.001(<0.001)
HGF	1.000±0.156	1.446±0.235	2.390±0.483	5.211±1.155	0.094	<0.001(0.001)	<0.001(<0.001)
IGF1	1.000±0.262	0.813±0.237	2.139±0.329	4.387±0.693	0.566	0.002(0.005)	<0.001(<0.001)
RORB	1.000±0.230	1.465±0.167	1.978±0.348	4.268±0.898	0.052	0.001(0.004)	<0.001(<0.001)
TNFSF10	1.000±0.282	0.643±0.081	2.259±0.499	3.961±0.548	0.052	<0.001(0.002)	<0.001(<0.001)
CTSL1	1.000±0.142	0.959±0.240	1.684±0.451	3.856±0.603	0.982	0.031(0.055)	<0.001(<0.001)
MGAT5	1.000±0.024	1.221±0.250	3.270±0.731	3.813±0.464	0.461	<0.001(<0.001)	<0.001(<0.001)
RPSA	1.000±0.159	0.744±0.187	1.756±0.138	3.664±0.637	0.132	0.004(0.010)	<0.001(<0.001)
FAT1	1.000±0.133	1.159±0.204	2.233±0.619	3.150±0.958	0.839	0.003(0.008)	<0.001(0.001)
TIMP2	1.000±0.335	1.276±0.099	1.517±0.109	3.130±0.447	0.212	0.025(0.046)	<0.001(<0.001)
MMP13	1.000±0.070	0.854±0.068	1.539±0.167	2.986±0.406	0.197	<0.001	<0.001(<0.001)
SYK	1.000±0.025	0.973±0.128	1.593±0.246	2.943±0.417	0.982	0.002(0.005)	<0.001(<0.001)
KRAS	1.000±0.259	1.210±0.211	1.954±0.263	2.858±0.367	0.419	0.001(0.003)	<0.001(<0.001)
VEGFA	1.000±0.214	1.063±0.347	1.612±0.281	2.406±0.619	0.952	0.025(0.046)	<0.001(0.001)
IL1B	1.000±0.215	1.300±0.132	2.313±0.510	2.754±0.623	0.290	0.001(0.002)	<0.001(0.001)
NR4A3	1.000±0.096	1.008±0.189	1.400±0.189	2.438±0.434	1.000	0.048(0.079)	<0.001(<0.001)
MMP11	1.000±0.481	1.170±0.643	1.284±0.141	1.261±0.229	0.184	0.018(0.021)	0.015(0.019)
KISS1R	1.000±0.043	1.205±0.250	1.555±0.211	2.158±0.331	0.413	0.007(0.017)	<0.001(<0.001)
DENR	1.000±0.239	1.461±0.327	2.345±0.412	2.089±0.499	0.121	0.001(0.002)	0.002(0.006)
MET	1.000±0.226	1.207±0.085	1.597±0.281	1.961±0.422	0.465	0.022(0.042)	0.002(0.005)
CXCL12	1.000±0.109	1.088±0.228	1.641±0.276	1.826±0.304	0.931	0.008(0.017)	0.002(0.005)
PNN	1.000±0.087	0.986±0.128	1.378±0.208	1.728±0.217	0.997	0.022(0.042)	<0.001(0.001)

### Effects of volatile anaesthetics on the expression of CXCR2, VEGFA, MMP11 and TGF -β1 on SKOV3 cells

**Figure 3:**
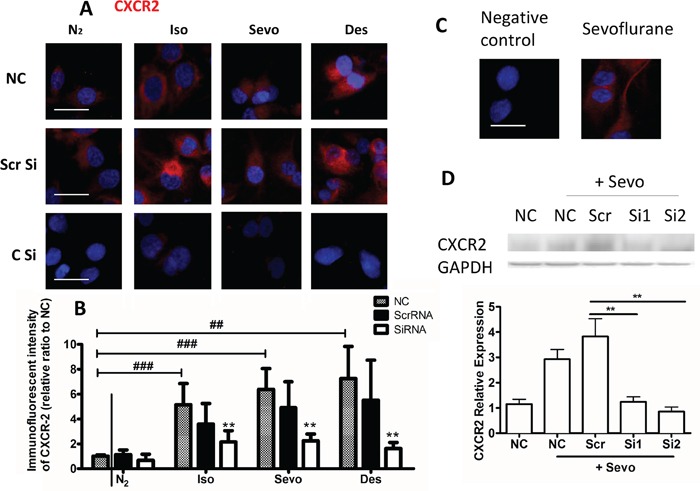
Expression of CXCR2 is increased in ovarian cancer upon exposure to volatile anaesthetics SK-OV3 cells were treated with air (N_2_) or 2% isoflurane (Iso) or 3.6% sevoflurane or 10.3% desflurane for 2 hours, and then recovered in the normal cell incubator for up to 24 hours. CXCR2 siRNA or scrambled siRNA was administered 6 hours before the gas exposure. Expression of **A.** CXCR2 (red) was assessed with immunofluorescent staining (nuclei counter-stained with DAPI) at 24 hour after gas exposure. Statistical analysis of fluorescent intensity of **B**. CXCR (n = 8). **C**. Negative control (secondary antibody with no primary antibody is added) demonstrated the specificity of the staining. **D**. Western blotting analysis of CXCR2 and GAPDH (n=4). Data are expressed as mean ± SD. *p<0.05 and ***p<0.001. Scale bar: 10μm. NC: naïve control. Iso: isoflurane. Sevo: sevoflurane, Des: desflurane. Scr Si: Scrambled siRNA, C Si: CXCR2 SiRNA. #: comparison between air and anaesthetic treated cells, *comparison between scrambled siRNA and CXCR2 siRNA treated cells.

**Figure 4:**
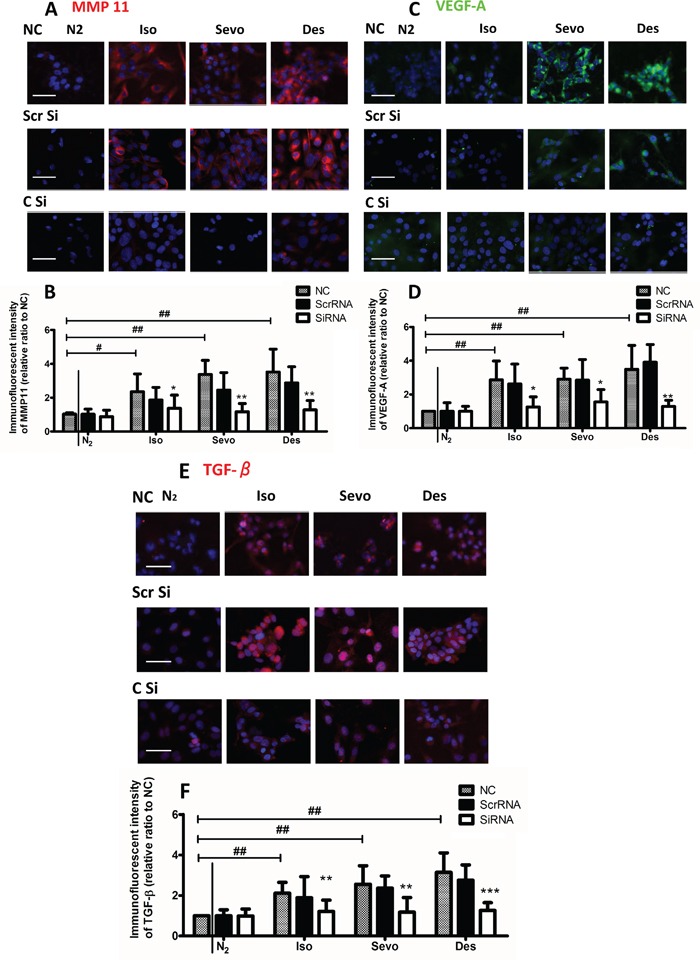
Upregulation of VEGF-A, MMP11 and TGF-β in ovarian cancer cells upon exposure to volatile anaesthetics SK-OV3 cells were treated with air (N_2_), 2% isoflurane (Iso) or 3.6% sevoflurane or 10.3% desflurane for 2 hours, and then recovered in the normal cell incubator for up to 24 hours. CXCR2 siRNA or scrambled siRNA was administered 6 hours before the gas exposure. Expression of **A**. MMP-11 (red), **C.** VEGF-A (red) and **E.** TGF-β (red) was assessed with immunofluorescent staining (nuclei counter-stained with DAPI) at 24 hour after gas exposure. Statistical analysis of fluorescent intensity of **B.** MMP-11, **D.** VEGF-A, and **F.** TGF-β (n = 8). Data are expressed as mean ± SD. *p<0.05 and ***p<0.001. Scale bar: 50μm. NC: naïve control. Iso: isoflurane. Sevo: sevoflurane, Des: desflurane. Scr Si: Scrambled siRNA, C Si: CXCR2 SiRNA. #: comparison between air and anaesthetic treated cells, *comparison between scrambled siRNA and CXCR2 siRNA treated cells.

Molecules such as VEGFA, MMP11 and TGF -β1 in particular CXCR2 play a very important role on cancer progression including ovarian cancer progression [13–18] and hence their expressions with or without siRNA after anesthetic exposure were further determined. The expression of CXCR2 protein after anaesthetic gas exposure was increased, compared with naive control (Figure 3A). CXCR2 has been shown to be overexpressed in ovarian cancer cell lines and to promote cancer metastasis [13] and so its role in anaesthetic-mediated effects on tumour biology was investigated: after transfection with CXCR2 siRNA, the post-exposure expression of CXCR, was all reduced significantly (Figure 3A and 3B). The negative control that was only probed with secondary antibody without primary antibody confirmed the specificity of the staining (Figure 3C), we have also assessed the transfection efficiency with western blot analysis and both siRNA transfection reduced the CXCR2 expression to the basal level (Figure 3D). Similarly, expression of MMP11, VEGF-A and TGF-β1 were enhanced by volatile anaesthetics and this effect was abolished after CXCR2 siRNA treatment (Figure 4).

### Effects of volatile anaesthetics on the cell migration of SK-OV3

**Figure 5:**
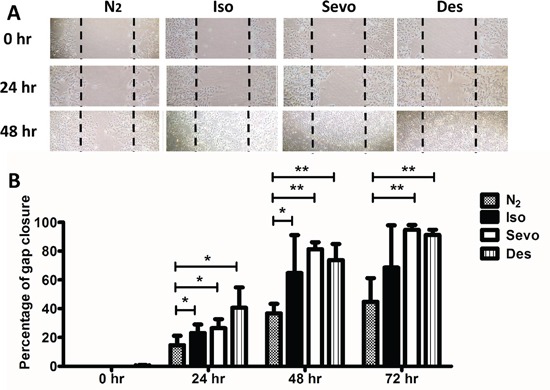
Migration of SKOV3 cells is increased after exposure to volatile anaesthetics SK-OV3 cells were treated with air (N_2_) or 2% isoflurane (Iso) or 3.6% sevoflurane or 10.3% desflurane for 2 hours, and then recovered in the normal cell incubator for up to 72 hours. CXCR2 siRNA or scrambled siRNA was administered 6 hours before the gas exposure. **A.** Cell migration at 0, 24, 48 and 72 hours after gas exposure, assessed by scratch assay (wound healing assay). **B.** % healing (gap closure) after gas exposure (n=8). Data are expressed as mean ± SD, *p<0.05, **p<0.01 and ***p<0.001. NC: naïve control. Iso: isoflurane, Sevo: sevoflurane, Des: desflurane.

Cell migration, assessed by gap closure, was significantly accelerated in the isoflurane, sevoflurane and desflurane groups compared to the control at 24 hours post-gas exposure, and at 48 hours the percentage of closure increased steadily in the anaesthetic groups whilst migration in the control group reached a plateau (Figure 5A and 5B). Significant differences were also observed at 72 hours post-gas exposure.

### CXCR2 siRNA treatment abolished the effects of volatile anaesthetics on the cell migration of SKOV3

**Figure 6:**
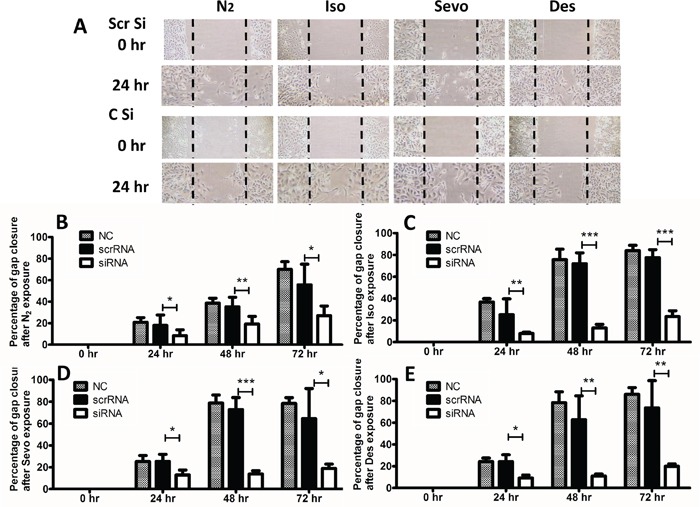
CXCR-2 siRNA abolished effects of inhalational anaesthetics on SK-OV3 cell migration SK-OV3 cells were treated with air (N_2_) or 2% isoflurane (Iso) or 3% sevoflurane or 10.3% desflurane for 2 hours, and then recovered in the normal cell incubator for up to 72 hours. **A.** Cell migration at 0, 24, 48 and 72 hours after gas exposure, assessed by scratch assay (wound healing assay). **B, C, D, E.** % healing (gap closure) after gas exposure (n=8). Data are expressed as mean ± SD, *p<0.05, **p<0.01 and ***p<0.001. NC: naïve control. Iso: isoflurane, Sevo: sevoflurane, Des: desflurane. Scr Si: Scrambled siRNA, C Si: CXCR2 SiRNA.

Based on the correlation between CXCR2 expression and the effects of volatile anaesthetics on cancer cells we carried out further investigations to evaluate the knock-down effect of CXCR2 on the migratory activity of SKOV3 cells (Figure 6A-6E). Cell migration in SKOV3 cells was dramatically suppressed upon transfection with CXCR2 siRNA in all anaesthetic groups compared to the NC and scrRNA groups. In the presence of CXCR2 siRNA, desflurane reduced gap closure by 60%, 62% and 38% relative to the scrRNA group at 24, 48 and 72 hours respectively (60%, 70% and 49% relative to the control group).

## DISCUSSION

This is the first study to systemically examine the change of metastatic gene profile of ovarian cancer cells after being exposed to three inhalational anesthetics: isoflurane, sevoflurane and desflurane. The findings in this study enhanced our understanding of the impact of volatile anaesthetics on cancer growth and metastasis and provided a novel insight into molecular mechanisms. Most importantly, our work supports clinical concern over current anaesthetic regimens for cancer patients[4].

Volatile anaesthetic agents are widely used in cancer surgery. Whilst their ability to alter the microenvironment and protein expression in healthy organs have been reported [7], to date only a few retrospective studies have indicated that cancer recurrence and metastasis after surgery may be linked to anaesthetic technique [3, 6]. It has been suggested that volatile anaesthetics may alter mRNA expression in cancer cells [19]. This study confirms that mRNA for CXCR2, VEGF-A, MMP11 and TGF-β mRNA's are all significantly increased after exposure to volatile anaesthetics, indicating the activation of key molecular mediators of metastasis such as cancer cell transformation, basement membrane degradation and angiogenesis.

Consistent with the profile of metastatic gene expression, our study clearly demonstrated the change of migration potential after exposure to inhalational anaesthetics. Our previous data obtained from prostate cancer cells [20], ovarian cancer cells [21] and renal carcinoma cells [22] indicated that isoflurane up-regulates the synthesis of HIF-1α via the PI3K/Akt/mTOR pathway and promotes the metastatic potential of cancer. CXCR2 has previously been shown to increase cell proliferation [23], cancer angiogenesis [13], and promote the resistance of cancer cells to conventional treatment [24]. Overexpression and binding of CXCL1 and its receptor CXCR2 has been shown to promote tumour invasion [25–27]. The present study confirms CXCR-2 as a novel therapeutic target to control anaesthetic mediated effects on ovarian cancer cell biology. Its central role was supported by the observation that genetic knockdown of CXCR-2 by siRNA abolished the promoting effects of inhalational anaesthetics on SK-OV3 migration. This is consistent with previous studies which showed that tumour growth and invasion was inhibited when CXCR2 is down-regulated [28, 29]. Also, some previous studies indicated a link between CXCR family and VEGF. In microenvironment of renal carcinoma cells, VEGF expression seemed to be correlated with CXCR expression [30]. Clinical outcomes were reported to be related to CXCR and VEGF expressions in esophagogastric cancer [31] and in lung carcinoma[32].

The loss of responsiveness to inhibitory growth signals exerted by TGF-β was previously established in ovarian cancer cells [33]. TGF-β has been demonstrated to enhance the aggressiveness of ovarian cancer cells [34]. All these evidence strongly support our conclusion that up-regulation of both CXCR2 and TGF-β promote the change of ovarian cancer towards a more invasive phenotype.

In this study, both array analysis and immunofluorescence data show significant increases in expression of MMP11 and VEGF-A which was suppressed after CXCR2 siRNA transfection, indicating an interaction between CXCR2 and MMP11. Matrix metalloproteinases (MMPs) are zinc-dependent enzymes capable of degrading extracellular matrix and basement membrane components and are involved in tumour metastasis [35]. MMP expression in tissue has been correlated with increased cancer cell proliferation and invasion [36]. MMP-11 is frequently found in ovarian carcinomas and is involved in ovarian cancer invasion [37]. MMP-11 overexpression has also been demonstrated to confer a poor prognosis in breast carcinomas [38] and was significantly correlated with the metastasis of hepatocellular carcinoma [39].

Angiogenesis has been established as a vital component in the mechanisms responsible for tumour metastasis[40]. Vascular endothelial growth factor (VEGF) is a well-known pro-angiogenetic factor[41], which stimulates neo-vascularisation and increases microvascular permeability[41, 42]. Higher levels of VEGF are frequently observed in ovarian carcinomas in patients [43] and it has been shown to play a critical role in different stages of ovarian cancer development and metastasis [44]. An interaction between MMP11 and VEGF-A has also been reported [39], and our study clearly demonstrated that inhalational anaesthetics promote the production of both MMP11 and VEGF-A, enhancing cell migration through basement membrane degradation and the formation of new vasculature.

Several study have suggested that inhalational anaesthetics may affect intracellular calcium homeostasis, especially via activation of InsP3R[45, 46]. While the intracellular calcium concentration is an important regulator of cell growth and death, signal transduction and hormone secretion, gene expression for proteins such as PI3K/Akt, MAPK, Erk has been shown to be involved[46]; however, the exact mechanism in cancer cells remains unclear, this certainly warrants further investigation in our future studies.

Compared with inhalational anaesthesia, Propofol, on the other hand, has been shown to be beneficial for the patients after cancer surgery, and studies have shown that propofol inhibited the invasion of cancer cells [47]. We have also demonstrated that isoflurane induced up-regulation of HIF-1α and its downstream effectors in prostate cell line. However, Propofol decreased HIF-1α accumulation induced by hypoxia or even isoflurane-induced HIF-1α activation, and partially reduced cancer cell malignant activities [20].

Our study is not without limitations. Firstly, compared to general anaesthesia, the use of regional anaesthesia has been shown to reduce recurrence rates in ovarian[6], breast [48] and prostate cancer[3]. However, the mechanisms by which tumour cells respond to different anaesthetics at a molecular level remain unclear. It would be interesting to compare the pattern of the change of genetic profile in cell culture exposed to general and local anaesthetics. Secondly, the cancer metastatic potential was assessed only in a cell culture model with one cell line, short time course and single siRNA. The kinetics of selected markers in longer time course remained unknown. Thirdly, the wound healing assay is an established standard protocol to analyse the cancer cell migration capability but undoubtedly cell proliferation is not exclusive to contribute the outcome. Lastly, there could be more anaesthetic effects on cancer cells with different dose or time courses, or even nonspecific effects of volatile agents. However, one can argue that the cancer cell biology changes observed in this study are very likely their “intrinsic” pharmacological effects in which it has been demonstrated previously that isoflurane effects on cancer cell growth and migration were concentration- and time-dependent [22]. Further *in vivo* studies should be considered for future investigation to verify these *in vitro* findings. Nevertheless, the work reported here provides potentially useful information on the effects of specific anaesthetics on cancer cell biology rather than simply suggesting a class effect.

## CONCLUSIONS

Together, our results suggest that volatile anaesthetics promote changes in the expression of metastatic genes and proteins. This study strongly suggests that inhalational anaesthetics have a distinct and profound effect on cancer growth and its capacity to metastasise. In addition, this study provides novel insights into molecular mechanisms and could lead to the identification of new anaesthetic regimens for cancer patients during surgery. However, this area of research into the potential impact of anaesthetics on cancer biology is just beginning, and extensive preclinical and clinical studies are warranted before any decisions can be made to change current clinical practice.

## MATERIALS AND METHODS

### Cell culture

Human ovarian epithelial carcinoma cell line SK-OV3 (European Cell Culture Collection) was used for this study. Cell cultures were kept at 37°C in a humidified atmosphere containing 5% CO2. SK-OV3 cells were cultured in McCoy's 5A medium (Sigma-Aldrich, Dorset, UK), containing 10% foetal bovine serum (Thermo Scientific, Epsom, UK), 2mM L-glutamine and 1% penicillin (Sigma-Aldrich, Dorset, UK) and maintained in a humidified incubator at 37°C with a 5% CO_2_ atmosphere.

### Test gas exposure

Cells were placed in a purpose-built 1.5L airtight gas chamber, equipped with inlet and outlet valves and an electric fan to ensure an even distribution of gases throughout the exposure period. All gases (BOC, South Humberside, UK) were delivered to the gas chamber at a rate of 2L/min for a maximum of 5mins until the desired gas and anaesthetic concentrations were achieved. The chamber gases were monitored using an anaesthetic analyser (Datex-Ohmeda, Stirling, UK) until the chamber was sealed. The chamber was then placed in an incubator (Galaxy R CO_2_ chamber; New Brunswick Scientific, Enfield, USA) at 37°C for the duration of the 2 hour incubation. The experimental gas mixtures were 21% oxygen, 5% carbon dioxide and 2% isoflurane, 3.6% sevoflurane or 10.3% desflurane, balanced with nitrogen. The equal 1.7 minimum alveolar concentrations (MAC) in human of these volatile anaesthetic concentrations was used in this study. After exposure, cultures were returned to the normal culture incubator for further study.

### RNA extraction

At 6 hours after gas exposure, total RNA was extracted from each 60mm dish using the RNeasy mini kit® and QIAshredder (QIAGEN, West Sussex, UK) according to the manufacturer's instructions. RNA quantity and quality were assessed using a BioPhotometer (Eppendorf, Stevenage, UK). Samples with an A260/A280 ratio above 1.8 were considered of sufficient quality for further analysis.

### Array

Array analysis was performed using a Tumour Metastasis PCR Array (Qiagen) according to the manufacturer's instructions. The RT^2^ First Strand Kit (Qiagen) was used to produce complementary DNA (cDNA) from total RNA. cDNA samples were mixed with RT^2^ SYBR® Green ROX qPCR (Qiagen) before loading into each well of the PCR Array. PCR array plates were processed and analysed with the 7900HT Fast Real-Time PCR system relative quantitation software (Applied Biosystems, LLC, Foster City, USA). mRNA expression relative to β-actin mRNA was determined using the comparative 2^−ΔΔC^_T_ (using online software provided by Qiagen) method and these values were subsequently converted into heat maps (relative levels of mRNA expression).

### qRT-PC

Gene expression was quantified using the Rotor-Gene Q system (Qiagen) in the presence of SYBR green (Qiagen). cDNA was mixed with master mix, forward and reverse primers each and of probe. Paired oligonucleotide primers were designed for vascular endothelial growth factor A (VEGF-A), matrix metalloproteinase 11 (MMP-11), transforming growth factor beta-1 (TGFβ1), chemokine (C-X-C motif) receptor 2 (CXCR2) and β-actin using Primer Designer (Scientific and Educational Software, Durham, USA) against the sequence downloaded from GenBank and were supplied by Invitrogen. The primer sequences, r^2^ values and efficiencies are summarised in Table 3. All TaqMan probes were supplied by Qiagen. All mRNA data were expressed relative to the endogenous control gene β-actin.

**Table 3:** Primer sequence for PCR

	Forward primer sequence	Reverse primer sequence	GenBank/EMBL association No.	Nucleotide No.	r2	efficiency
Actin β	5′-AGAGCTACGA GCTGCCTGAC-3′	5′-AGCACTGTGT TGGCGTACAG-3′	NM_001101.3	797-980	0.99979	0.98
CXCR2	5′-ACATGGGCAA CAATACAGCA-3′	5′-TGAGGACGAC AGCAAAGATG-3′	NM_001557.3	1014-1193	1.00000	0.94
MMP 11	5′-GACGGACCTC ACCTACAGGA-3′	5′-CAGTACCTGGC GAAGTCGAT-3′	NM_005940.3	343-510	0.99998	0.94
TGF β1	5′-CAACAATTCCT GGCGATACC-3′	5′-GAACCCGTTGA TGTCCACTT-3′	NM_000660.4	1407-1599	0.99981	1.24
VEGFA	5′-CTACCTCCACC ATGCCAAGT-3′	5′-CACACAGGATG GCTTGAAGA-3′	NM_001171630.1	1086-1272	0.99982	0.92

### In vitro siRNA transfection

Cells were seeded in 6-well plates at 60-80% confluency, allowed to settle for 24 hours and then transfected with scrambled siRNA (ScrRNA) or two different CXCR2 siRNA constructs (siRNA1: Sense Strand 5′**→** 3′- CAGUCAGGAUUUAAGUUUATT; 5′**→** 3′- UAAACUUAAAUCCUGACUGGG. siRNA2: 5′**→** 3′- CCUCAAGAUUCUAGCUAUATT; 5′**→** 3′- UAUAGCUAGAAUCUUGAGGAG) (Qiagen, Crawley, West Sussex, UK) at a concentration of 20nM. The transfection was facilitated with HiPerfect Transfection Reagent (Qiagen). After 6 hours of transfection, the solution was replaced with medium before exposure to test gas mixtures. Then cells treated with siRNA1 or with both siRNA 1 and 2 will be used for *in situ* immunostaning and western blot respectively (see below).

### Scratch assay (wound healing) assay

Cells were seeded into 60mm Petri dishes and subsequently scratched before exposure to the test gas mixture, after which cells were incubated for 24 hours. The scratch assay (wound healing assay) was performed for assessing tumour cell migration as previously described[49]. One artificial gap per well was scratched into the monolayers with a sterile plastic 1000μL micropipette tip to generate a uniform gap that was devoid of adherent cells. Cells were washed with PBS to remove cell debris. Closure of the scratch was monitored using an inverted microscope at 4x objective and digital camera (Olympus CK30-SLP, Japan) at 0, 24, 48 and 72 hours after gas exposure and analysed using Image J 1.46 software (National Institute of Health, Maryland, USA). Gap closure (healing) was quantified as the mean percentage of the remaining cell-free area compared with the area of the initial wound[50].

### Immunostaining

Cells were fixed in 4% paraformaldehyde for 10mins, rinsed in PBS (Sigma-Aldrich, Dorset, UK) two times for 5min, permeabilised with 0.1% Triton X-100 in PBS (PBS-T) for 10min and washed twice for 5min. Blocking was carried out at room temperature for 1hr using 10% normal donkey serum. Cells were then incubated overnight at 4°C in blocking solution containing one of the following antibodies: mouse anti- VEGF-A (1:200, Abcam plc, Cambridge, United Kingdom), rabbit anti-MMP-11 (1:200, Abcam plc, Cambridge, United Kingdom), rabbit anti-TGF-β (1:200, Abcam plc, Cambridge, United Kingdom) and rabbit anti–CXCR2 (1:200, Abcam plc, Cambridge, United Kingdom) followed by Rhodamine-conjugated or fluorescein isothiocyanate (FITC)-conjugated secondary antibody incubation (Millipore, Watfield, UK). Finally, the coverslips were mounted onto glass slides with Vectashield mounting medium containing DAPI (Millipore). Cells were viewed under the Olympus BX60 (Olympus, Hamburg, Germany) wide-field fluorescence microscope under a 20X objective using the appropriate filter. Images were captured using a cooled Zeiss AxioCam camera with Zeiss software. Images for quantification were pre-processed using Image J 1.46 software background subtraction (National Institutes of Health, Maryland, USA). All images were captured at identical exposure settings. Cells were measured by manual tracking and the mean pixel intensity was calculated.

### Western blot

This was done with our established protocol[20]. Briefly, after electrophoresis and transferred onto a polyvinylidenedifluoride (PVDF) membrane using the iBlot® 2 Dry Blotting System (Invitrogen). Membranes were blocked with 5% non-fat powdered milk in Tris-Buffered Saline with Tween (TBS-T) for 1h at room temperature, and then incubated overnight at 4°C with anti-CXCR2 rabbit primary antibody (Abcam, 1:500) followed by horseradish peroxidase (HRP)-linked anti-rabbit secondary antibody (Cell Signaling; 1:1000) for 1 hour. Protein bands were visualised using the enhanced chemiluniscence (ECL) system (Santa Cruz, USA) and the Syngene GeneSnap software (Syngene, UK). Densitometry analysis was carried out using the corresponding GeneTools software (Syngene) and presented as a ratio of the protein of interest to a control housekeeping protein for analysis.

### Statistical analysis

All numerical data is expressed as mean ± SD. One-way ANOVA analysis followed by post-hoc Tukey's test were applied for most of the analysis using Prism ver5.0 (GraphPad Software, Inc., California, USA), unless otherwise specified. To validate array data, the results from array and single qRT-PCR analysis of mRNAs were examined using the paired t-test. For analysing our array results, the false discovery rate was used as described as described previously[51], the false discovery rate (q-value) was set at 0.1 using the program QVALUE 2.0 (http://genomics.princeton.edu/storeylab/qvalue/). P values of less than or equal to 0.05 were taken to indicate statistical significance.
